# The impact of systemic treatment on brain metastasis in patients with non-small-cell lung cancer: A retrospective nationwide population-based cohort study

**DOI:** 10.1038/s41598-019-55150-6

**Published:** 2019-12-10

**Authors:** Jung Soo Lee, Ji Hyung Hong, Der Sheng Sun, Hye Sung Won, Yeo Hyung Kim, Mi Sun Ahn, Seok Yun Kang, Hyun Woo Lee, Yoon Ho Ko

**Affiliations:** 1 Department of Rehabilitation Medicine, Uijeongbu St. Mary’s Hospital, College of Medicine, The Catholic University of Korea, Seoul, Republic of Korea; 2 Division of Oncology, Department of Internal Medicine, Incheon St. Mary’s Hospital, College of Medicine, The Catholic University of Korea, Seoul, Republic of Korea; 3 Division of Oncology, Department of Internal Medicine, Uijeongbu St. Mary’s Hospital, College of Medicine, The Catholic University of Korea, Seoul, Republic of Korea; 4 Department of Hematology-Oncology, Ajou University School of Medicine, Suwon, Republic of Korea; 5 Cancer Research Institute, College of Medicine, The Catholic University of Korea, Seoul, Republic of Korea

**Keywords:** Non-small-cell lung cancer, Non-small-cell lung cancer

## Abstract

To compare the incidence of brain metastases of advanced non-small cell lung cancer (NSCLC) treated with systemic cytotoxic chemotherapy (CC) and targeted therapy (TT), we performed a large-scale, retrospective, nationwide, cohort study. The population data were extracted from the Health Insurance Review and Assessment Service of Korea database from January 1, 2011, to November 30, 2016. Of the 29,174 patients newly diagnosed with stage IIIB or IV NSCLC who received systemic treatment, we investigated the initial and subsequent incidence of brain metastases. Besides, among 22,458 patients without initial brain metastasis, the overall cumulative incidence of subsequent brain metastases was compared according to systemic treatment administered. In total, 1,126 (5.0%) patients subsequently developed brain metastasis. The overall cumulative incidence of brain metastasis was significantly higher in the TT group than in the CC group (1-year cumulative incidence: 8.7% vs. 3.8%; 3-year: 17.2% vs. 5.0%; P < 0.001). Younger age, female sex, and first-line TT were significant risk factors for subsequent brain metastasis. In conclusion, the overall cumulative incidence of brain metastasis was significantly higher in patients received TT as the first-line treatment than in those received CC.

## Introduction

Lung cancer is the leading cause of cancer-related death, and has a dismal prognosis. Most lung cancers are non-small cell lung cancer (NSCLC)^1^. Approximately 20–40% of patients with NSCLC develop brain metastasis, which is a serious complication with disabling neurological symptoms that decrease quality of life, and has a dismal prognosis^2,3^. Therefore, an enhanced understanding of the pattern of brain metastasis in NSCLC patients would contribute the treatment strategies resulting in improvements in quality of life of patients.

The histology and molecular features of NSCLC, such as epidermal growth factor receptor (*EGFR*) mutation and anaplastic lymphoma kinase (*ALK*) translocation, affect the clinical prognosis. In previous reports, among Korean patients with stage IV NSCLC, 18.1% showed brain metastasis at the time of diagnosis; this was more common in patients with a mutated *EGFR* (27.4%) than wild-type *EGFR* (14.5%)^4^. Cytotoxic chemotherapy (CC) plays a limited role in the treatment of brain metastasis because of the agents’ inability to cross the blood–brain barrier (BBB)^5^. By contrast, EGFR or ALK tyrosine kinase inhibitors (TKIs) are more effective in controlling and preventing brain metastasis in NSCLC patients^6^. Therefore, identifying NSCLC brain metastasis and determining its relationship with genetic mutations and systemic therapy in the Korean population would enable the treatment strategies to be refined.

To date, no large-scale studies have assessed the development of brain metastases according to systemic CC and targeted therapy (TT) in NSCLC patients. Thus, using the Health Insurance Review and Assessment Service of Korea (HIRA) data, we investigated the incidence of brain metastasis in patients with advanced NSCLC who received palliative systemic treatment and compared the cumulative incidence of brain metastasis according to the systemic treatment administered.

## Materials and Methods

### Study design

**Figure 1:**
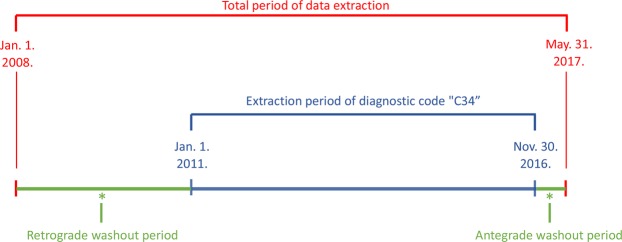
Health Insurance Review and Assessment Service of Korea data used to identify patients with newly diagnosed non-small-cell lung cancer (NSCLC).

This large-scale, retrospective, nationwide, cohort study was approved by the institutional review board of the Uijeongbu St. Mary Hospital and HIRA (No. UC18ZESI0001). The study protocol was performed in accordance with the guidelines and regulation of the institutional review board of the Uijeongbu St. Mary Hospital. The requirement for written informed consent was waived because of this study’s character of retrospective analysis. All medical service billing records of 446,601 patients having lung cancer (ICD-10 code C34) in between January 1 2008 and May 31 2017 were offered from the database of HIRA (Fig. 1).

### Study population

**Figure 2:**
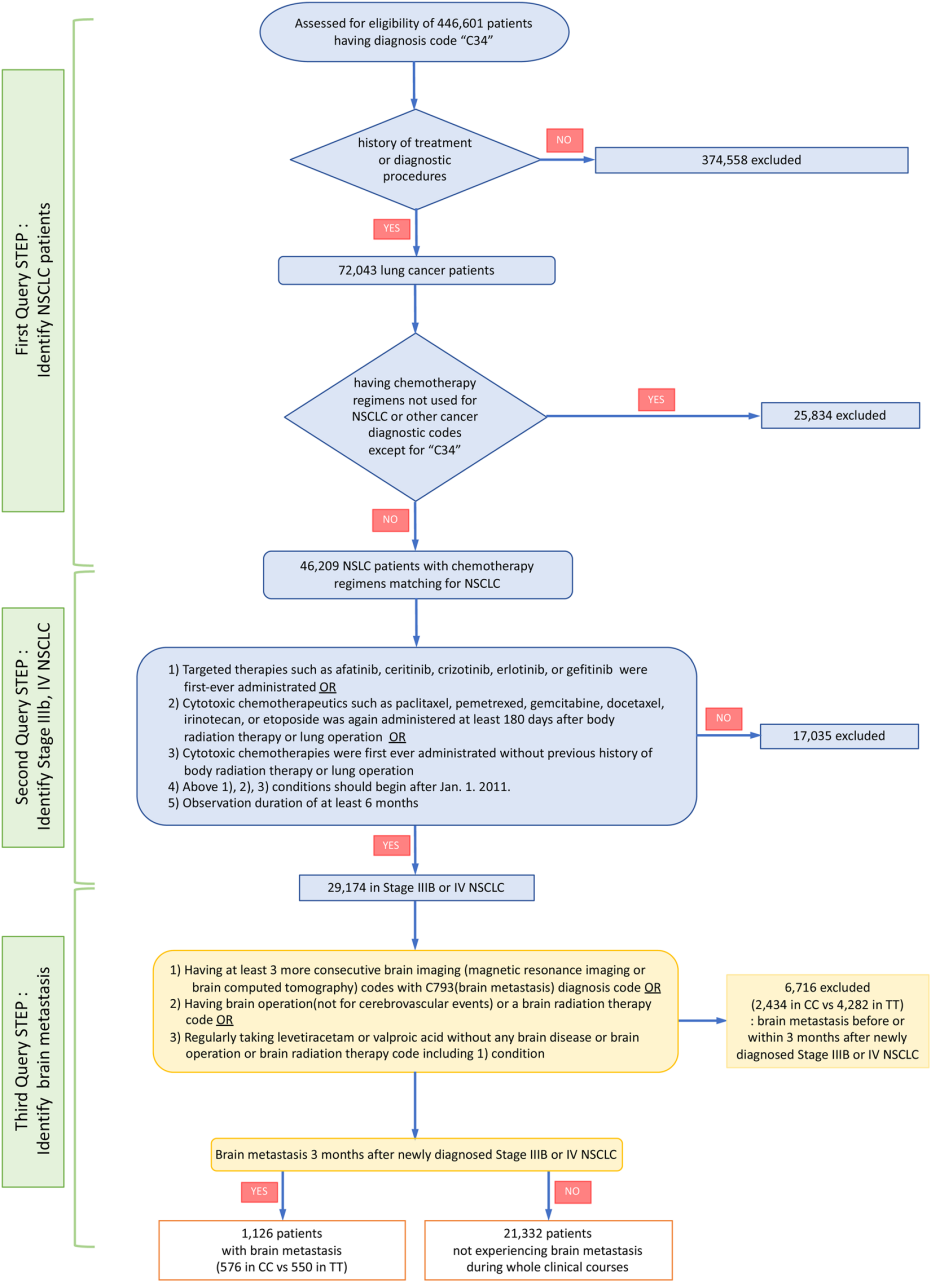
Data mining to identify patients newly diagnosed with stage IIIB or IV NSCLC without brain metastasis. Patients in the CC and TT groups received cytotoxic chemotherapy and targeted therapy, respectively, as the first-line treatment.

The data-mining scheme used in this study is shown in Fig. 2. Being compulsory, Korean health insurance covers the entire population, HIRA provides information on medical fees and assesses the quality of healthcare services provided to the Korean population. Thus, HIRA data includes all ICD-10 diagnostic codes and billing codes of all medical services, such as diagnostic procedures, treatment modalities (such as drug prescriptions, radiotherapy or surgery), provided to the entire population of Korea.

To select the appropriate patient cohort, we performed several queries. In the first query, to discriminate newly diagnosed NSCLC patients, the billing codes of treatment and drugs in 446,601 patients with a C34 ICD-10 code were queried. The treatment billing codes consisted of lung surgery and radiotherapy (body or brain), and the drugs prescribed were paclitaxel, pemetrexed, gemcitabine, docetaxel, erlotinib, afatinib, ceritinib, crizotinib, and gefitinib. In total, 72,043 patients were identified using the above queries, of which 25,834 were filtered out as they had received chemotherapy not used for NSCLC or had other types of cancer. The remaining 46,209 patients were selected as newly diagnosed NSCLC patients between January 1 2011 and Nov 30 2016.

We regarded patients who underwent lung surgery as having early stage NSCLC, those who received radiotherapy of the lung parenchyma as having locally advanced NSCLC, and patients who received only systemic CC or TT appropriate for NSCLC as having metastatic or recurrent disease. To verify the survival outcome reliability of NSCLC patients, a survival analysis was performed according to treatment pattern. A Kaplan–Meier analysis showed the median survival duration and 5-year survival rate to be 75.6 months (95% confidence interval [CI], 71.4–81.1) and 57.19 ± 0.72%, 26.3 months (95% CI, 25.4–27.3) and 26.01 ± 0.66%, and 13.1 months (95% CI, 12.9–13.3) and 11.56 ± 0.23% in 46,209 patients received operation, lung radiotherapy, and systemic chemotherapy, respectively (Supplemental Fig. 1). These findings are comparable with those of a recent study of NSCLC patients^7^.

Next, to identify patients with stage IIIB or IV NSCLC the following queries were used: (1) first-ever administration of targeted therapeutics such as afatinib, ceritinib, crizotinib, erlotinib, or gefitinib; OR (2) cytotoxic chemotherapeutics such as paclitaxel, pemetrexed, gemcitabine, docetaxel, irinotecan, or etoposide administered at least 180 days after radiotherapy or surgery on the lung parenchyma; OR (3) first-ever administration of cytotoxic chemotherapeutics and no history of radiation therapy or surgery on the lung parenchyma; (4) conditions (1), (2), and (3) began after January 1 2011; and (5) observation duration of at least 6 months. In total, 29,174 patients were found to have been diagnosed with stage IIIB or IV NSCLC and received palliative chemotherapy.

In the third query, to identify patients with brain metastasis of newly diagnosed stage IIIB or IV NSCLC, the following operational definitions were used: (1) at least three consecutive brain imaging (magnetic resonance imaging or computed tomography) codes with brain metastasis (ICD-10, C793) in the absence of a history of treatment for brain metastasis or cerebrovascular events, OR (2) having brain surgery not for cerebrovascular events or a brain radiation therapy code, OR (3) regularly taking anticonvulsant drugs such as levetiracetam or valproic acid without a code for brain disease or brain surgery or brain radiation therapy, plus condition (1). The diagnostic codes and billing codes of all treatments and drugs are available in HIRA homepage (http://www.hira.or.kr).

In total, 6,716 patients with brain metastasis identified before or less than 3 months after the initial diagnosis with stage IIIB or IV NSCLC were excluded. Finally, 22,458 stage IIIB or IV NSCLC patients without brain metastasis initially were analyzed. Among them, patients who received CC as the first-line treatment were defined as the CC group, and patients who received targeted therapy (TT) were defined as the TT group. Because the HIRA database does not include information on genetic mutations, the patients in the TT group were regarded as having a mutation in *EGFR* or *ALK*. Brain metastasis at 3 months or more after diagnosis with stage IIIB or IV NSCLC was defined as subsequent brain metastasis, and brain metastasis less 3 months after diagnosis was defined as initial brain metastasis.

### Definition of survival outcomes

The overall survival (OS) duration was calculated from the date of diagnosis to the date of death or the last follow-up visit. The date of diagnosis was defined as the date when first chemotherapy or surgery or radiotherapy was started after the first-ever application of the C34 diagnostic code. The date of death was extracted from the insurance dataset. If the date of death could not be identified and there were no verified records of visits to the outpatient or inpatient clinic for 6 months before May 31 2017, the patient was regarded as dead.

### Statistical analysis

Baseline characteristics are presented as means (±standard error) and medians (ranges) for continuous variables and frequencies (%) for categorical variables. A *t*-test was performed for comparisons of continuous variables and Pearson’s chi-squared test or two-sample proportion z-test for comparisons of categorical variables. Kaplan-Meier method was performed to estimate the probability of brain metastasis (CC or TT failure) or survival. Unfortunately, the proportional hazards assumption was violated in this study. Poisson regression analysis, a type of generalized linear models, was performed to estimate relative risk (RR). The SAS Enterprise Guide version 6.1 (SAS Inc., Cary, NC, USA), Visual Basic for Applications 7.0 (Microsoft Inc., Redmond, WA, USA), and Excel 2010 (Microsoft Inc., Redmond, WA, USA) was used to perform data mining and statistical analyses.

## Results

### Incidence of brain metastasis in stage IIIB or IV NSCLC patients

Among 29,174 patients with newly diagnosed stage IIIB or IV NSCLC, 7,847 (26.9%) experienced brain metastasis. 6,716 (23.0%) (2,434 and 4,282 in the CC and TT groups, respectively) had initial brain metastasis. Among 1,126 patients with subsequent brain metastasis, 576 and 550 were in the CC and TT groups, respectively. The incidence of initial and subsequent brain metastasis was higher in the TT group than in the CC group (P < 0.0001); however, the time to brain metastasis development did not differ significantly between the TT and CC groups (P = 0.082) (Supplemental Table 1).

### Baseline characteristics

Finally, 22,458 patients were selected for analysis of subsequent brain metastasis. Their demographic features are listed in Table 1. Of the patients, 16,119 (71.8%) received TT as their first-line treatment, while 6,339 (28.2%) received CC. Within an 8.6-month median follow-up period (range, 0–77.5 months), 1,126 (5.0%) patients developed subsequent brain metastasis.

**Table 1:** Demographic characteristics of 22,458 patients with stage IIIB or IV NSCLC without initial brain metastasis.

	Total patients (n = 22,458, 100%)	Patients with subsequent BM (n = 1,126, 5.0%)	Patients without subsequent BM (n = 21,332, 95.0%)	*P*-value
**Age**	67.23 ± 10.31	61.87 ± 10.86	67.51 ± 10.20	<0.0001
**Sex (M/F)**	14,555/7,903	493/633	14,062/7,270	<0.0001
Medical condition				
HBP	11,202 (49.9%)	487 (43.3%)	10,715 (50.2%)	<0.0001
DM	4,706 (21.0%)	195 (17.3%)	4,511 (21.1%)	<0.0001
COPD	896 (4.0%)	22 (2.0%)	874 (4.1%)	<0.0001
Anticoagulation user	542 (2.4%)	31 (2.8%)	511 (2.4%)	<0.0001
Antiplatelet user	6,535 (29.1%)	278 (24.7%)	6,257 (29.3%)	<0.0001
First line treatment				
Cytotoxic chemotherapy	16,119 (71.8%)	576 (51.2%)	15,543 (72.9%)	<0.0001
Targeted therapy	6,339 (28.2%)	550 (48.8%)	5,789 (27.1%)	

Abbreviations: BM, brain metastasis; HBP, hypertension; DM, diabetes mellitus; COPD, chronic obstructive pulmonary disease.

### Factors associated with subsequent brain metastasis

Younger age, female, and absence of chronic obstructive pulmonary disease (COPD) were identified as risk factors for subsequent brain metastasis. In addition, the use of TT compared to that of CC as a first-line treatment was a significant risk factor, likely because NSCLC patients with genetic mutations have a higher incidence of subsequent brain metastasis. After adjusting for all variables at baseline, the associations of the above risk factors with brain metastasis, except COPD, remained significant (Table 2).

**Table 2:** Relative risk of subsequent brain metastasis of stage IIIB or IV NSCLC patients.

	Unadjusted RR (95% CI)	*P*-value	Adjusted RR (95% CI)	*P*-value
Age				
<**30** vs. 40–49	1.869 (1.610–2.169)	<0.0001	2.105 (1.815–2.445)	**<0**.**0001**
<**30** vs. 50–59	2.907 (2.494–3.401)	<0.0001	2.994 (2.570–3.496)	**<0**.**0001**
<**30** vs. 60–69	4.049 (3.322–4.926)	<0.0001	3.846 (3.165–4.695)	**<0**.**0001**
<**30** vs. ≥70	3.690 (2.525–5.405)	<0.0001	3.521 (2.421–5.128)	<0.**0001**
Sex (**female** vs. male)	2.364 (2.110–2.653)	<0.0001	1.821 (1.600–2.070)	**<0**.**0001**
Anticoagulation (**non-user** vs. user)	0.873 (0.860–1.236)	0.445	0.905 (0.642–1.277)	0.571
COPD (**absence** vs. presence)	2.083 (1.376–3.165)	0.001	1.170 (0.769–1.779)	0.465
First-line systemic treatment (**TT** vs. CC)	2.427 (2.169–2.717)	<0.0001	1.976 (1.745–2.242)	**<0**.**0001**

Abbreviations: RR, relative risk; CI, confidence interval; CC, cytotoxic chemotherapy; TT, targeted therapy; COPD, chronic obstructive pulmonary disease.

### Overall cumulative incidence of subsequent brain metastasis according to systemic treatment

**Figure 3:**
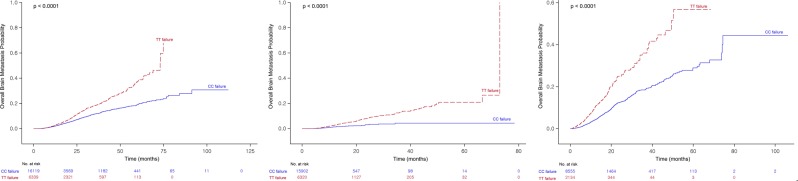
Overall cumulative incidence of subsequent brain metastasis according to first-line therapy during the whole observation period (**A**), during first-line treatment (**B**), and after first-line treatment failure (**C**). Patients in the CC and TT groups received cytotoxic chemotherapy and targeted therapy, respectively, as the first-line treatment.

The overall cumulative incidence of subsequent brain metastasis was significantly higher in those who received TT than in those who received CC as the first-line treatment (1-year cumulative incidence, 2.4 ± 0.2% *vs*. 1.9 ± 0.2%; 3-year cumulative incidence, 18.6 ± 0.9% *vs*. 11.9 ± 0.6%; P < 0.0001; Fig. 3A).

Notably, the overall cumulative incidence of brain metastasis during the initial 10 months of first-line treatment did not differ between two groups (Fig. 3B). However, after 10 months of first-line treatment, the overall cumulative incidence of brain metastasis was higher in the patients who received TT until the start of second-line treatment (TT *vs*. CC; 1-year cumulative incidence, 2.8 ± 0.3% *vs*. 1.4 ± 0.2%; 2-year cumulative incidence, 8.2 ± 0.7% *vs*. 3.3 ± 0.6%; P < 0.0001; Fig. 3B).

Next, we analyzed whether brain metastasis occurred during or after the second-line treatment period among patients who did not have brain metastasis during first-line treatment. The cumulative incidence of brain metastasis during or after second-line treatment was higher in *EGFR/ALK* TKI-failed patients than in CC-failed patients (TT *vs*. CC; 1-year incidence, 10.3 ± 0.9% *vs*. 4.2 ± 0.3%; 3-year, 37.0 ± 3.1% *vs*.18.6 ± 1.0%; respectively, P < 0.0001; Fig. 3C).

The cumulative incidence of brain metastasis during first-line treatment did not differ according to the first-line *EGFR* TKI applied (P = 0.283; Supplemental Fig. 2A). The cumulative incidence of brain metastasis during or after second-line treatment was significantly higher in the gefitinib-failed patients than in the erlotinib-failed patients (P = 0.081; Supplemental Fig. 2B).

### Factors associated with survival outcomes

Patients who developed subsequent brain metastasis had a higher RR for survival than those who did not (unadjusted RR, 1.089 (1.125–1.053), P < 0.0001; adjusted RR, 1.250 (1.208–1.294), P < 0.0001; Table 3). Patients in the TT group (median OS duration, 14.9 months; 95% CI, 14.3–15.4; 5-year OS rate, 11.6%) had significantly better survival outcomes compared to those in the CC group (median OS duration, 6.8 months; 95% CI, 6.6–7.0 months; 5-year OS rate, 9.2%, P < 0.0001) (Supplemental Fig. 3).

**Table 3:** Relative risk for overall survival of 22,458 patients.

	Unadjusted RR (95% CI)	*P*-value	Adjusted RR (95% CI)	*P*-value
Age				
<**30** vs. 40–49	0.921 (0.904–0.939)	<0.0001	**0**.**890 (0**.**907–0**.**875)**	**<0**.**0001**
<**30** vs. 50–59	0.837 (0.816–0.861)	<0.0001	**0**.**827 (0**.**806–0**.**849)**	**<0**.**0001**
<**30** vs. 60–69	0.812 (0.774–0.853)	<0.0001	**0**.**821 (0**.**784–0**.**861)**	**<0**.**0001**
<**30** vs. ≥70	0.773 (0.695–858)	<0.0001	**0**.**779 (0**.**702–0**.**864)**	**<0**.**0001**
Sex (**female** vs. male)	0.802 (0.787–0.818)	<0.0001	**0**.**907 (0**.**888–0**.**925)**	**<0**.**0001**
Anticoagulation (**non-user** vs. user)	1.072 (1.009–1.139)	0.024	0.957 (0.983–1.044)	0.159
COPD (**absence** vs. presence)	0.888 (0.858–0.918)	<0.0001	0.988 (0.955–1.779)	0.465
Brain metastasis (**presence** vs. absence)	1.089 (1.125–1.053)	<0.0001	**1**.**250 (1**.**208–1**.**294)**	**<0**.**0001**
First-line systemic treatment (**TT** vs. CC)	0.669 (0.653–0.686)	<0.0001	**0**.**686 (0**.**668–0**.**704)**	**<0**.**0001**

Abbreviations: RR, relative risk; CI, confidence interval; CC, cytotoxic chemotherapy; TT, targeted therapy; COPD, chronic obstructive pulmonary disease.

Brain metastasis, older age, male sex, anticoagulant use, COPD and first-line CC were identified as risk factors for poor survival outcomes. The effect of risk factors remained significant even after adjusting for baseline variables, except anticoagulant use and COPD.

## Discussion

We investigated the incidence of brain metastasis in NSCLC patients who received CC and TT as the first-line treatment. The overall prevalence of brain metastasis was 26.9% among stage IIIB or IV NSCLC patients who received palliative systemic treatment; of these patients without initial brain metastasis, 5.0% subsequently developed brain metastasis. Because of the characteristics of the HIRA database and the coverage by national insurance of first-line EGFR TKIs only for patients with *EGFR* mutations after 2011 in South Korea, patients who received TT as their first-line treatment were regarded as having genetic aberration. The overall cumulative incidence of brain metastasis was significantly higher in patients regarded as having genetic aberrations than in those without them. In addition to the first-line TT, younger age, and female sex were risk factors for subsequent brain metastasis.

NSCLC patients harboring *EGFR* mutations are reported more likely to have brain metastases^4,8–14^. In our study, the overall incidence of brain metastasis among patients in the TT group (regarded as having aberrations of *EGFR/ALK genes*) was 32.7%, compared to 20.1% in the CC group (regarded as having wild-type *EGFR/ALK genes*). These rates are similar to the 31.4% and 19.7% of Japanese patients with mutated and wild-type *EGFR*, and the 37.0% and 21.5% of Korean patients, respectively^8,11,12^. Similarly, the frequency of *initial* brain metastasis was higher in the TT (27.4%) group than in the CC group (17.9%). In accordance with our results, the incidence of initial brain metastasis in *EGFR* mutation patients is reportedly 19–27%, compared to 9–22% in *EGFR* wild-type patients^4,9,10^. Moreover, the cumulative incidence of subsequent brain metastasis was significantly higher in the TT group (5.3%) than in the CC group (2.2%), which is also comparable to previous studies^4^. Notably, the presence of the *EGFR* exon19 or L858R point mutations, which accounted for 90% of the *EGFR* mutations, is predictive of subsequent brain metastasis^13^. *In vitro*, overexpression of mutated *EGFR* induces morphological changes toward a mesenchymal phenotype and promotes the mobility of lung cancer cells^13^, implying that NSCLC patients with *EGFR* mutations have a higher probability of distant metastasis than do those with wild-type *EGFR*. Thus, first-line TT usage, which means the patients presumed to have gene aberrations, was a risk factor for initial and subsequent brain metastasis.

First-generation EGFR TKIs such as gefitinib and erlotinib show marked intracranial activity against the development and progression of brain metastasis in patients with mutated *EGFR*^15–19^. The combined analysis of LUX-Lung 3 and 6 trials, afatinib, a second-generation EGFR TKI significantly improved progression-free survival in patients with NSCLC plus brain metastases and common EGFR mutations (8.2 *vs*. 5.4 months; hazard ratio, 0.50)^20^. In a retrospective study, NSCLC patients who responded to EGFR TKIs showed the 1- and 2-year CNS failure rates of 14% and 36%^21^. In our study, the 1- and 3-year cumulative incidences were 2.4% and 18.6%, respectively. However, treatment modalities of brain metastases and the characteristics of the study subjects differed among each study. Thus, well-designed large-scale observational cohort studies are needed to clarify the patterns of brain metastasis in NSCLC patients with *EGFR/ALK* aberrations.

After failure of the first-line treatment, the incidence of brain metastasis increased in the TT group compared to the CC group. Acquired resistance mutations and the flare phenomenon likely explain such rapid progression^22^. This increase of brain metastasis was frequently observed in the patients who received gefitinib as the first-line treatment than in those who received erlotinib. The serum concentration of TKIs is important to overcome their incomplete BBB penetration to effectively control cancer cells with *EGFR* mutations in the brain^23^. The approved dose of erlotinib is determined as its maximum tolerated dose (MTD), whereas gefitinib is used at approximately one-third of its MTD^24^. Inefficient elimination of *EGFR*-mutant cancer cells by gefitinib’ lower CNS concentration than erlotinib’s may explain the higher incidence of brain metastases after failure of gefitinib compared to erlotinib.

The risk factors for subsequent brain metastasis were younger age, female sex, as well as TT usage as the first-line treatment. In the Surveillance, Epidemiology, and End Results data, the incidence of brain metastasis is higher in female patients and those <60 years of age^25^. Females with lung cancer are more likely than males to be never-smokers, have adenocarcinoma with an activating *EGFR* or *ALK* mutation, which increase the risk of distant metastasis.

Several limitations to the present study are worth mentioning. There may have been bias in the stratification according to the clinical stage, molecular features or in the definition of brain metastasis. Because the patients’ data was derived from the HIRA database and ICD-10 code, the information regarding stage, the molecular features, the presence of brain metastasis, the progress of the disease and causes of death had to be inferred by the treatment pattern. However, to overcome any such bias we used a strict multistep approach for selection of appropriate patient cohort.

In conclusion, our results imply that the overall cumulative incidence of brain metastasis was significantly higher in patients received TT, regarded as having *EGFR/ALK* mutations, than in those not having *EGFR/ALK* mutations, although TT effectively inhibits the development of brain metastasis until 10 months of first-line treatment. In patients with *EGFR/ALK* mutations after TT failure, regular screening for brain metastasis and its treatment with agents that can penetrate the BBB and overcome resistance are needed.

## Supplementary information

Supplemental table and figures

## Data Availability

The datasets generated during and/or analysed during the current study are not publicly available due to Data Protection Laws and Regulations in Korea, but final analyzing results are available from the corresponding author on reasonable request.
