# Supplementary material for: The impact of systemic treatment on brain metastasis in patients with non-small-cell lung cancer: A retrospective nationwide population-based cohort study
Source: Sci Rep. 2019 Dec 10;9:18689. doi: 10.1038/s41598-019-55150-6 (PMC6904708; doi:10.1038/s41598-019-55150-6)

Supplementary data

The impact of systemic treatment on brain metastasis in patients with non-small-cell lung cancer: A retrospective nationwide population-based cohort study

JS Lee[1], JH Hong[2], DS Sun[3], HS Won[3], YH Kim[1], MS Ahn[4], SY Kang[4], HW Lee[4*], YH Ko[3,5*]

[1]Department of Rehabilitation Medicine, Uijeongbu St. Mary's Hospital, College of Medicine, The Catholic University of Korea, Seoul, Republic of Korea

[2]Division of Oncology, Department of Internal Medicine, Incheon St. Mary's Hospital, College of Medicine, The Catholic University of Korea, Seoul, Republic of Korea

[3]Division of Oncology, Department of Internal Medicine, Uijeongbu St. Mary's Hospital, College of Medicine, The Catholic University of Korea, Seoul, Republic of Korea

[4]Department of Hematology-Oncology, Ajou University School of Medicine, Suwon, Republic of Korea

[5]Cancer Research Institute, College of Medicine, The Catholic University of Korea, Seoul, Republic of Korea

Supplemental Table 1. Incidence of initial and subsequent brain metastasis according to systemic treatment (n = 29,174)

	CC group* (n = 13,564)	TT group† (n = 15,610)	Total (n = 29,174)	*P*-value
Within 3 months of diagnosis‡	2,434 (17.9%)	4,282 (27.4%)	6,716 (23.0%)	0.082[1]
More than 3 months after diagnosis¶	576 (2.2%)	550 (5.3%)	1,126 (3.9%)	
Brain metastasis, N (incidence, %)	3,010 (20.1%)	4,382 (32.7%)	7,847 (26.9%)	<0.0001[2]

*CC group: Patients who received CC as the first-line treatment were regarded as having no EGFR/ALK aberration.

† TT group: Patients who received TT as the first-line treatment were regarded as having EGFR/ALK aberration.

‡ Patients developed brain metastasis within 3 months of diagnosis of stage IIIB or IV NSCLC.

¶ Patients developed brain metastasis more than 3 months after diagnosis of stage IIIB or IV NSCLC.

[1]Pearson's chi-squared test.

[2]Two-sample proportion z-test.

Supplemental Figure 1. Survival curve of newly diagnosed NSCLC patients who underwent lung surgery (blue line) *vs*. radiation therapy (red line) *vs*. palliative chemotherapy only (green line).

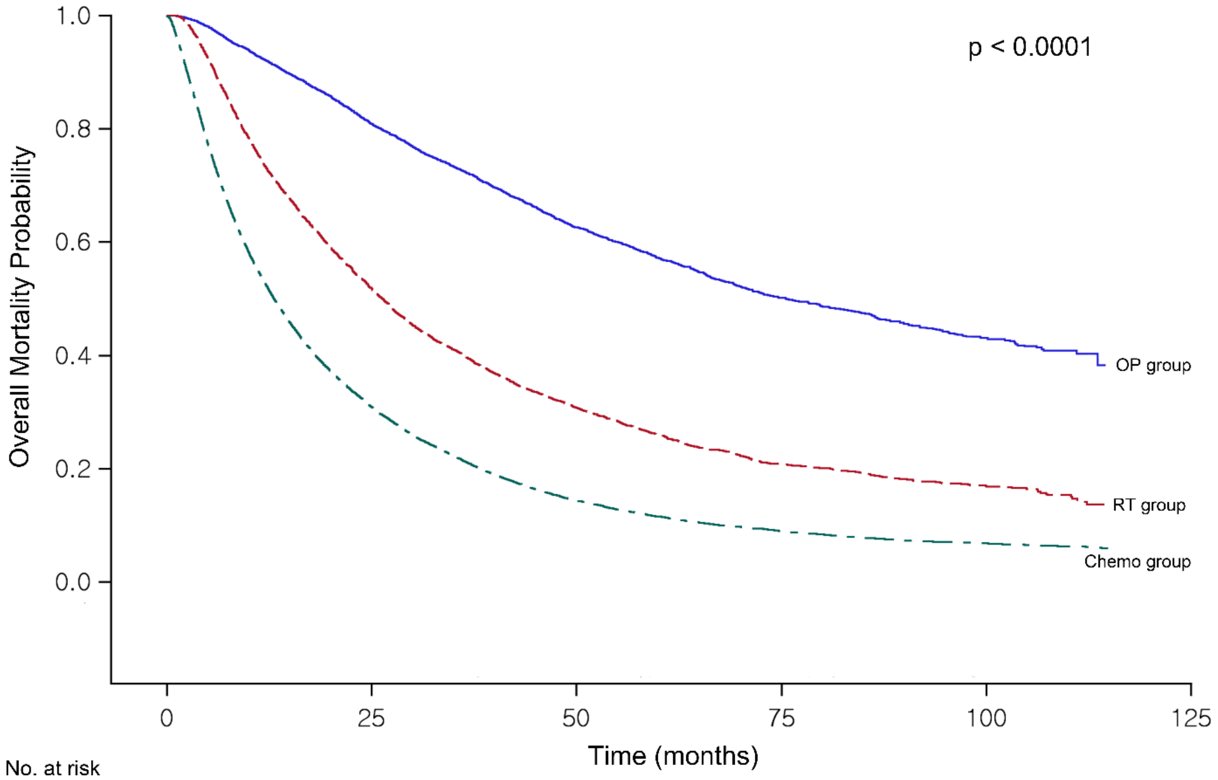

No. at risk

OP group	7297	5217	3180	1820	932	333	0
RT group	6097	3205	1566	788	377	166	0
Chemo group	32815	9271	3479	1553	741	333	0

Supplemental Figure 2A. Overall cumulative incidence of subsequent brain metastasis in NSCLC patients according to the epidermal growth factor receptor (EGFR) - tyrosine kinase inhibitor (TKI) administered as the first-line treatment during the observation period.

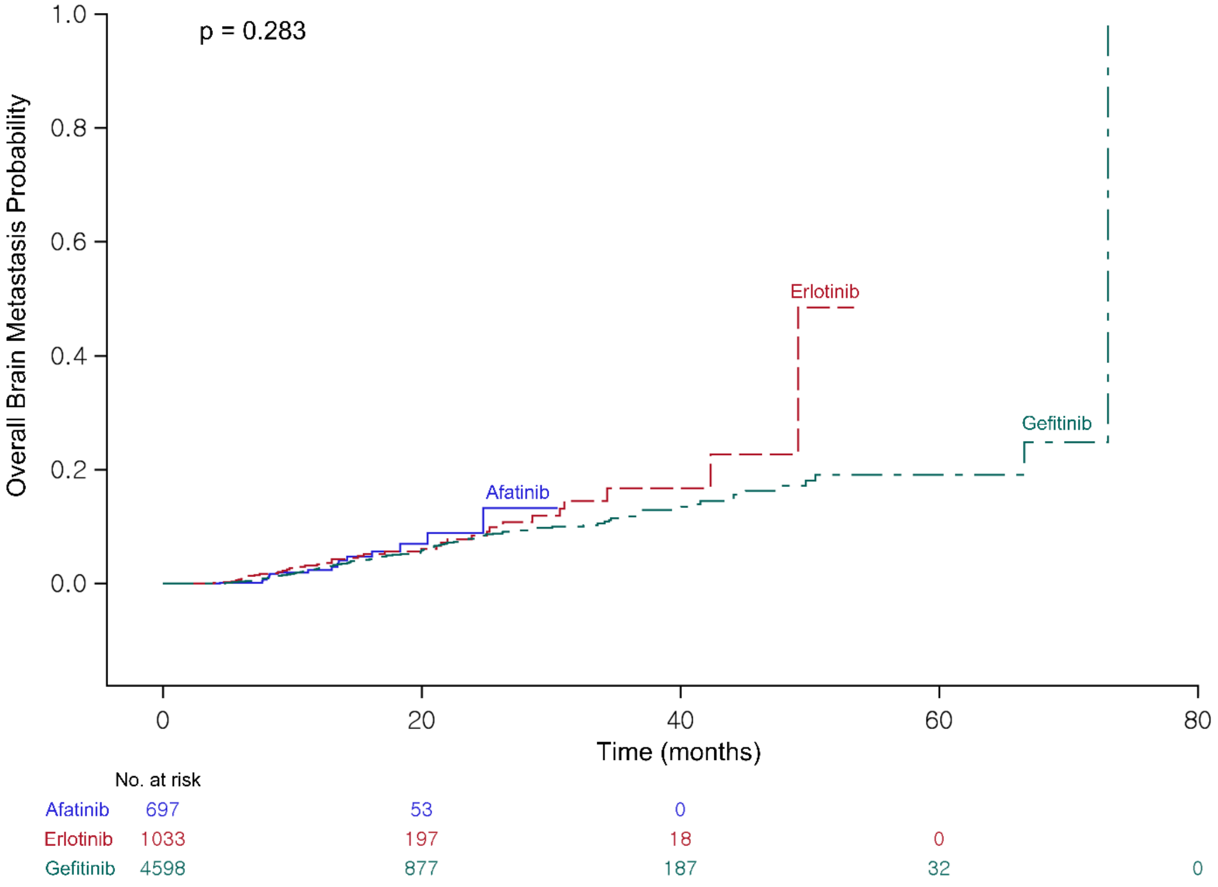

Supplemental Figure 2B. Overall cumulative incidence of subsequent brain metastasis in NSCLC patients according to the epidermal growth factor receptor (EGFR) - tyrosine kinase inhibitor (TKI) administered as the first-line treatment after second-line treatment following failure of the first-line EGFR-TKI.

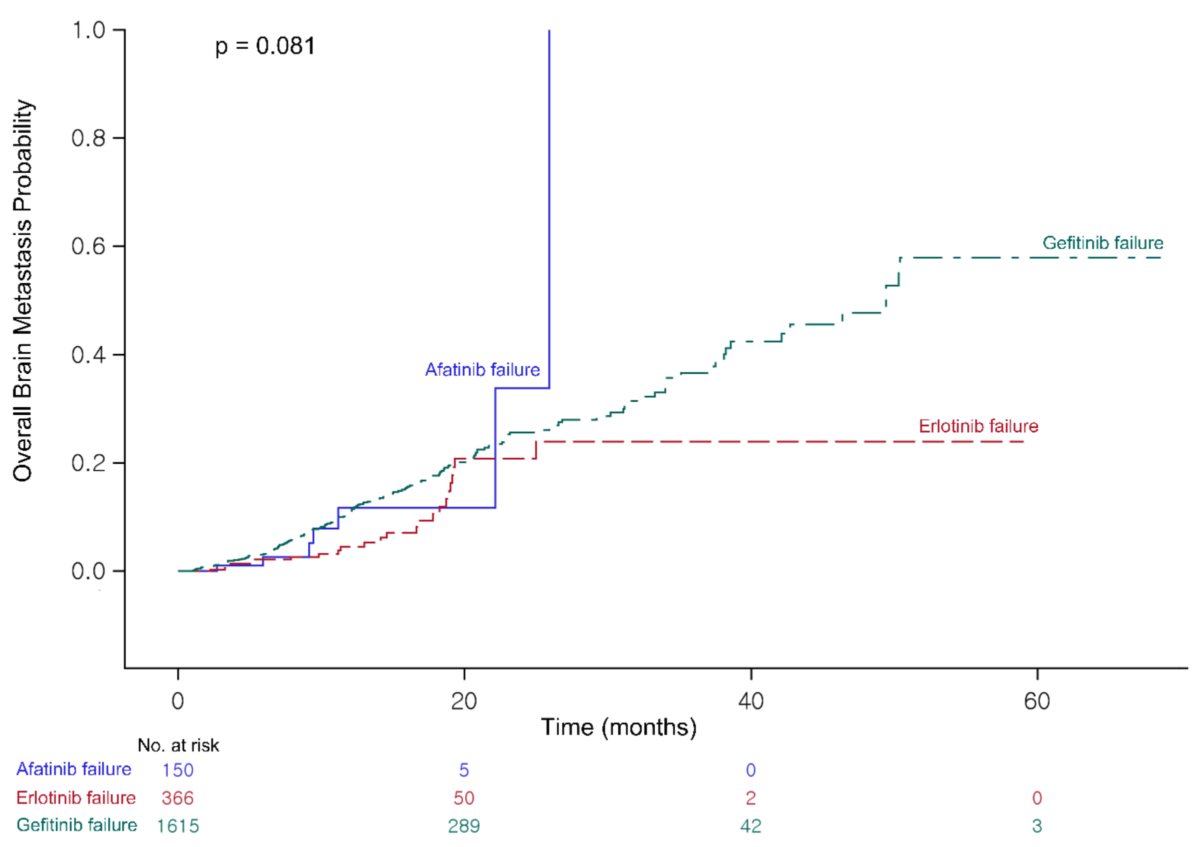

Supplemental Figure 3. Overall survival of NSCLC patients according to first-line treatment with CC or TT during the observation period. CC, cytotoxic chemotherapy; TT, targeted therapy.

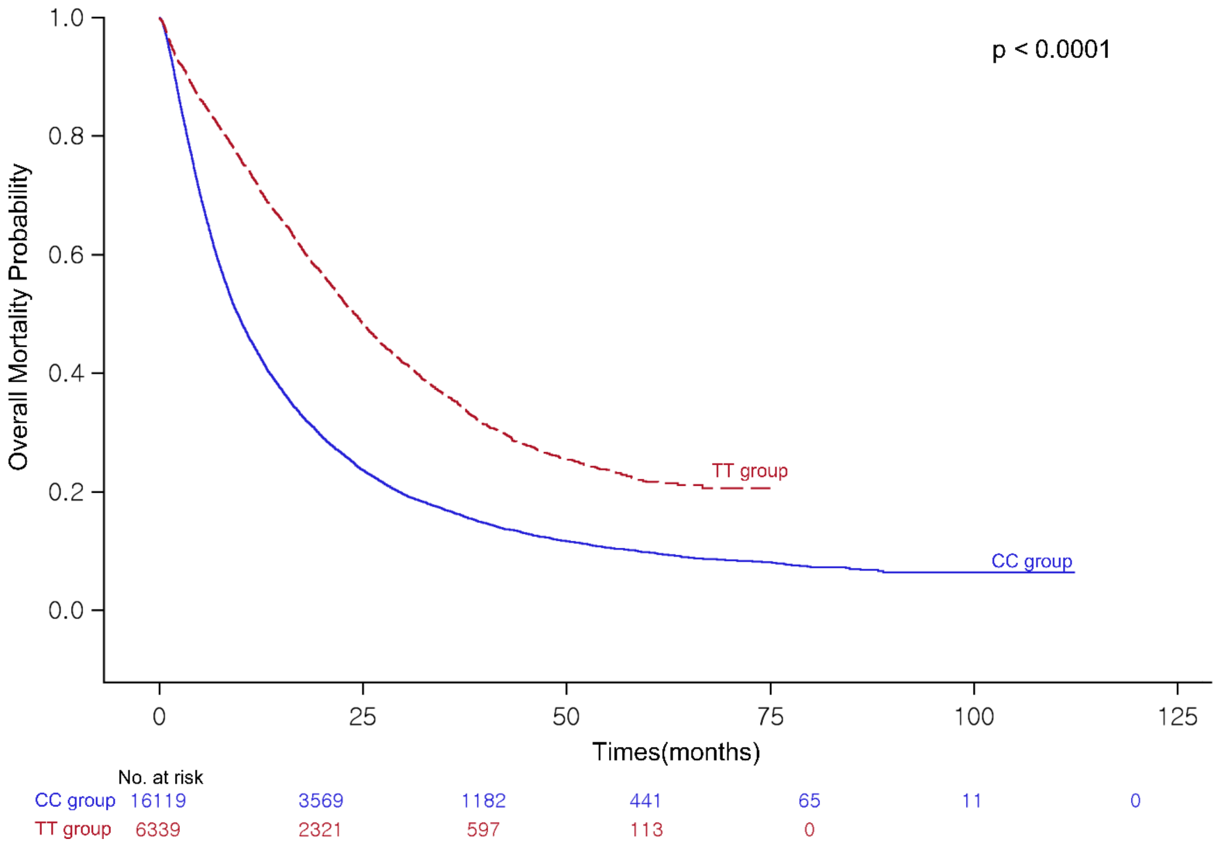

Supplement: Supplementary file 1 — Supplemental table and figures [file 41598_2019_55150_MOESM1_ESM.pdf]
